# Chemical coding of zinc-enriched neurons in the intramural ganglia of the porcine jejunum

**DOI:** 10.1007/s00441-012-1486-5

**Published:** 2012-08-24

**Authors:** Joanna Wojtkiewicz, Maciej Równiak, Robert Crayton, Mariusz Majewski, Sławomir Gonkowski

**Affiliations:** 1 Faculty Medical Sciences, Department of Neurology and Neurosurgery, Stem Cell Research Laboratory, University of Warmia and Mazury, ul. Warszawska 30, 10-082 Olsztyn, Poland; 2 Department of Comparative Anatomy, Faculty of Biology, University of Warmia and Mazury, Olsztyn, Poland; 3 Department and Clinic of Urology, Faculty of Medical Sciences, Medical University of Warsaw, Warsaw, Poland; 4 Department of Human Physiology, Faculty of Medical Sciences, University of Warmia and Mazury, Olsztyn, Poland; 5 Department of Clinical Physiology, Faculty of Veterinary Medicine, University of Warmia and Mazury, Olsztyn, Poland

**Keywords:** Zinc-enriched neurons, Jejunum, Small intestine, Immunolabeling technique, Enteric nervous system, Domestic pig

## Abstract

Zinc ions in the synaptic vesicles of zinc-enriched neurons (ZEN) seem to have an important role in normal physiological and pathophysiological processes in target organ innervation. The factor directly responsible for the transport of zinc ions into synaptic vesicles is zinc transporter 3 (ZnT3), a member of the divalent cation zinc transporters and an excellent marker of ZEN neurons. As data concerning the existence of ZEN neurons in the small intestine is lacking, this study was designed to disclose the presence and neurochemical coding of such neurons in the porcine jejunum. Cryostat sections (10 mμ thick) of porcine jejunum were processed for routine double- and triple-immunofluorescence labeling for ZnT3 in various combinations with immunolabeling for other neurochemicals including pan-neuronal marker (PGP9.5), substance P (SP), somatostatin (SOM), vasoactive intestinal peptide (VIP), nitric oxide synthase (NOS), leu-enkephalin (LENK), vesicular acetylcholine transporter (VAChT), neuropeptide Y (NPY), galanin (GAL), and calcitonin-gene related peptide (CGRP). Immunohistochemistry revealed that approximately 39%, 49%, and 45% of all PGP9.5- positive neurons in the jejunal myenteric (MP), outer submucous (OSP), and inner submucous (ISP) plexuses, respectively, were simultaneously ZnT3^+^. The majority of ZnT3^+^ neurons in all plexuses were also VAChT-positive. Both VAChT-positive and VAChT-negative ZnT3^+^ neurons co-expressed a variety of active substances with diverse patterns of co-localization depending on the plexus studied. In the MP, the largest populations among both VAChT-positive and VAChT-negative ZnT3^+^ neurons were NOS-positive cells. In the OSP and ISP, substantial subpopulations of ZnT3^+^ neurons were VAChT-positive cells co-expressing SOM and GAL, respectively. The broad-spectrum of active substances that co-localize with the ZnT3^+^ neurons in the porcine jejunum suggests that ZnT3 takes part in the regulation of various processes in the gut, both in normal physiological and during pathophysiological processes.

## Introduction

Recent studies on the chemical coding of the enteric nervous system (ENS) have identified a subpopulation of neurons that express the zinc transporter 3 (ZnT3; Gonkowski et al. 2009; Gonkowski 2011). ZnT3 is a member of the SLC 30 zinc transporter family, which is responsible for transport between extracellular, cytoplasmic, and intracellular organelle compartments (Palmiter and Huang 2004). Importantly, the ZnT3-mediated transport of zinc into synaptic vesicles serves to modulate neuron activity (Palmiter et al. 1996) and thus plays an important role in the normal physiological and pathophysiological changes of the nervous system (Frederickson et al. 2000, 2005; Smart et al. 2004). Zinc-enriched nerve (ZEN) terminals that utilize ZnT3 have been found in the hippocampus, amygdala, neocortex, spinal cord, and superior cervical ganglion neurons (Jo et al. 2000; Wang et al. 2003; Wenzel et al. 1997). Since previous studies on the central nervous system (CNS) have suggested that ZnT3 is present in neurons that use Zn as neuromodulator (Cousins et al. 2006; Danscher et al. 2003; Kim et al. 2000; Wang et al. 2003; Wenzel et al. 1997), ZnT3 can therefore be used as a marker for tracing ZEN structures. Although the role of Zn as a neuromodulator/neurotransmitter in the nervous system remains obscure, convincing evidence suggests that this cation is a potent modulator of various receptors and several transporters in the brain, and that it thereby influences both excitatory and inhibitory neurotransmission (Betz and Laube 2006; Frederickson et al. 2005; Smart et al. 2004). The presence of Zn in the ENS might suggest a similar modulatory role.

In humans and pigs, the ENS is organized into three plexuses: myenteric plexus (MP), outer submucous plexus (OSP), and inner submucous plexus (ISP). All these plexuses are populated with above 20 classes of neurons with different functions and targets and a large variety of neurotransmitters (Brown and Timmermans 2004; Furness 2000). Until now, only two studies have reported the presence of ZnT3 in the ENS. However, both of these studies have centered on the large intestine (Gonkowski et al. 2009; Gonkowski 2011). At present, no data on other parts of the gastrointestinal tract are available. Therefore, the aim of the present study has been to investigate the distribution, number, and chemical coding pattern of ZnT3^+^ neurons in the intramural ganglia of the porcine jejunum.

## Materials and methods

### Study subjects

Six juvenile female pigs (8–10 weeks, 12–15 kg body weight) of the Large White Polish breed were used. All animals were housed and treated in accordance with the Principles of Laboratory Animal Care (NIH publication no. 86–23, revised 1985). All experimental procedures were approved by the Local Ethics Commission of the University of Warmia and Mazury in Olsztyn (no. 27/2009).

### Anesthesia and surgery

All animals were pretreated with atropine sulfate (Polfa, Poland; 0.04 mg/kg body weight, s.c.) and azaperone (Stressnil, Janssen Pharmaceutica, Belgium; 2.0 mg/kg body weight, i.m.) 30 min prior to administration of the main anesthetic. Surgery was performed under fractionated thiobarbital (Thiopenthal, Sandoz, Austria; 20 mg/kg b.w., i.v.) anesthesia. All animals were killed by overdoses of thiobarbital and perfused transcardially with 4% buffered paraformaldehyde (pH 7.4). Following perfusion, the jejunum from all the animals was dissected out, cut into tissue blocks, postfixed by immersion in the same fixative for 4 h, washed twice in 0.1 M phosphate buffer (*pH* 7.4, 4°C) for 3 days, and then stored in 18% sucrose at 4°C until sectioned. Other organs from these animals were also collected for further research in our or other laboratories.

### Immunofluorescence experiments

Jejunum samples were cut into sections (10 μm thick) by using a cryostat (Hyrax C25; Carl Zeiss, Germany) and processed for double- and triple-immunofluorescence. All samples were washed three times in phosphate-buffered saline (PBS) and then incubated in humid chambers with blocking buffer (0.1 M PBS, 10% normal horse serum, 0.01% bovine serum albumin, 1% Tween, 0.05% thimerosal, 0.01% NaN_3_) for 1 h. The sections were then rinsed in PBS and incubated overnight at room temperature with a mixture of primary antibodies, namely a combination of the appropriate primary antisera to zinc transporter 3 (ZnT3), pan-neuronal marker (PGP9.5), substance P (SP), somatostatin (SOM), vasoactive intestinal peptide (VIP), nitric oxide synthase (NOS), leu-enkephalin (LENK), vesicular acetylcholine transporter (VAChT), neuropeptide Y (NPY), galanin (GAL), and calcitonin-gene related peptide (CGRP; Table 1). After incubation with the primary antibodies, the sections were rinsed in PBS and incubated for 1 h with biotinylated secondary antibodies (during double-labeling immunofluorescence) or with biotinylated 7-amino-4-methylcoumarin-3-acetic acid (AMCA; during triple-labeling immunofluorescence; Table 1). After 1 h, the sections were finally incubated with a mixture of fluorescein isothiocyanate (FITC) and CY3-conjugated streptavidin (Table 1). Finally, all samples were rinsed in PBS and then mounted with carbonate-buffered glycerol (pH 8.6) and coverslipped.

**Table 1:** Specification of immune reagents vs. zinc transporter 3 (*PGP9.5* pan-neuronal marker, *ZnT3* zinc transporter 3, *NOS* nitric oxide synthase, *VIP* vasoactive intestinal peptide, *SP* substance P, *SOM* somatostatin, *LENK* leu-enkephalin, *VAChT* vesicular acetylcholine transporter, *NPY* neuropeptide Y, *GAL* galanin, *CGRP* calcitonin-gene related peptide, *FITC* fluorescein isothiocyanate, *AMCA* 7-amino-4-methylcoumarin-3-acetic acid, *H* heavy chain, *L* light chain)

Antisera	Code	Host species/specificity	Dilution	Supplier
Primary antibody				
PGP9.5	7863-2004	Mouse	1:2000	Biogenesis, UK;www.biogenesis.co.uk
ZnT3	–	Rabbit	1:600	Gift from Prof. Palmiter, USA
NOS	N2280	Mouse	1: 2000	Sigma, US; www.sigma-aldrich.com
VIP	9535-0504	Mouse	1: 2000	Biogenesis
SP	8450-0505	Rat	1:300	
SOM	8330-0009	Rat	1: 100	
LENK	4140-0355	Mouse	1: 1000	
VAChT	H-V007	Goat	1: 2000	Phoenix, Pharmaceuticals, US; www.phoenixpeptide.com
NPY	NZ1115	Rat	1:300	Biomol Research Laboratories, US
GAL	T-5036	Guinea pig	1:1000	Peninsula Labs, US; see Bachem; www.bachem.com
CGRP	T-5027	Guinea pig	1:1000	
Secondary antibodies				
FITC-conjugated IgG (H + L)	715-095-151	Donkey-anti-mouse	1:800	Jackson
	712-095-153	Donkey-anti-rat	1:800	
	706-095-148	Donkey-anti-guinea pig	1:1000	
	705-096-147	Donkey-anti-goat	1:1000	
Biotinylated IgGs	E 0432	Goat anti-rabbit	1:1000	DAKO, E 0432
Biotin conjugated F(ab)’ fragment of affinity-purified IgG (H + L)	711-1622	Anti-rabbit	1:1000	BioTrend, 711-1622
AMCA-conjugated IgG (H + L)	715-155-151	Donkey-anti-mouse	1:50	Jackson
	715-155-153	Donkey-anti-rat	1:50	
	705-156-147	Donkey-anti-goat	1:50	
CY3-conjugated streptavidin	016-160-084	-	1:9000	

### Controls

Standard controls, i.e., preabsorption for the neuropeptide antisera (20 mg appropriate antigen per 1 ml corresponding antibody at working dilution) and the omission and replacement of all primary antisera by non-immune sera or PBS, were applied to test both antibody and method specificity.

### Counts and statistics

The sections were observed by using an Olympus BX51 fluorescence microscope equipped with epi-fluorescence and an appropriate filter set for FITC, CY3, and AMCA. Microphotographs were acquired with Cellsens Olympus image analysis software (ver. 3.2; Soft Imaging System, Münster, Germany). To determine the percentages of ZnT3^+^ neurons in each plexus studied (MP, OSP, and ISP), pan-neuronal marker PGP-9.5 was adopted. PGP-9.5 marks all neurons in the tissue, and so PGP^+^/ZnT3^+^ cells illustrate the percentages of MP, OSP, and ISP neurons co-expressing ZnT3. At least 1000 PGP9.5-labeled cell bodies located in 60–70 ganglia of a particular plexus (MP, OSP, and ISP) per animal were examined for ZnT3 immunoreactivity. Only neurons with clearly visible nuclei were counted. To prevent double-counting of ZnT3^+^ neurons, the sections were located at least 200 μm apart from each other. Moreover, to determine the percentages of co-localization of ZnT3 with other substances studied, at least 700 ZnT3-positive cell bodies in particular types of enteric plexuses were examined for immunoreactivity to the particular substances investigated. In these double- and triple-labeling studies, ZnT3-positive neurons were considered as representing 100% for all combinations, and so all the values shown in the text and Table 2 are percentages of ZnT3^+^ neurons. Finally, data were pooled from all 6 animals and expressed as means±SD and then analyzed by using GraphPad Prism 5 software (GraphPad Software, La Jolla, Calif., USA).

**Table 2:** Neurochemical characterization of zinc-transporter-3-immunoreactive (*ZnT3* ^*+*^) neurons in the enteric ganglia of the porcine jejunum (*MP* myenteric plexus, *OSP* outer submucosal plexus, *ISP* inner submucosal plexus, *s* single neurons). Note that PGP9.5 is a pan-neuronal marker that marks all neurons in the tissue, and so PGP^+^/ZnT3^+^ cells illustrate the percentages of MP, OSP, and ISP neurons co-expressing ZnT3. In the triple-labeling studies, ZnT3-positive neurons were considered as representing 100% for all combinations with other neurotransmitters, and so all the values presented are percentages (means±SD) of ZnT3^+^ neurons

Labeling	MP	OSP	ISP
PGP^+^/ZnT3^+^	38.6±4.0	48.7±10.8	44.8±4.1
ZnT3^+^/VAChT^−^	39.2±5.6	5.8±1.2	2.8±1.0
ZnT3^+^/VAChT^−^/^−^NOS^+^	37.7±5.4	4.1±1.8	0
ZnT3^+^/VAChT^−^/VIP^+^	5.8±3.4	1.3±0.9	2.1±0.9
ZnT3^+^/VAChT^−^/SOM^+^	5.4±1.2	2.2±0.8	1.1±0.8
ZnT3^+^/VAChT^−^/SP^+^	2.5±1.3	0.9±0.5	1.5±0.7
ZnT3^+^/VAChT^−^/LENK^+^	1.2±0.6	0	0
ZnT3^+^/LENK^+^/SP^+^	s	0	0
ZnT3^+^/VAChT^−^/GAL^+^	0	s	2.0±0.8
ZnT3^+^/VAChT^−^/NPY^+^	s	0	0
ZnT3^+^/VAChT^−^/CGRP^+^	0	0	0
ZnT3^+^/VAChT^+^	60.8±5.6	94.2±1.2	97.2±4.3
ZnT3^+^/VAChT^+^/SOM^+^	2.2±1.2	41.1±1.8	27.2±5.4
ZnT3^+^/VAChT^+^/VIP^+^	1.1±0.3	21.9±1.3	30.9±2.3
ZnT3^+^/VAChT^+^/SP^+^	2.5±1.3	16.1±2.8	28.4±1.9
ZnT3^+^/VAChT^+^/GAL^+^	0	0	47.1±1.9
ZnT3^+^/VAChT^+^/NOS^+^	10.4±3.1	3.7±1.1	0

## Results

### Distribution and number of ZnT3^+^ neurons in porcine jejunum

**Fig. 1:**
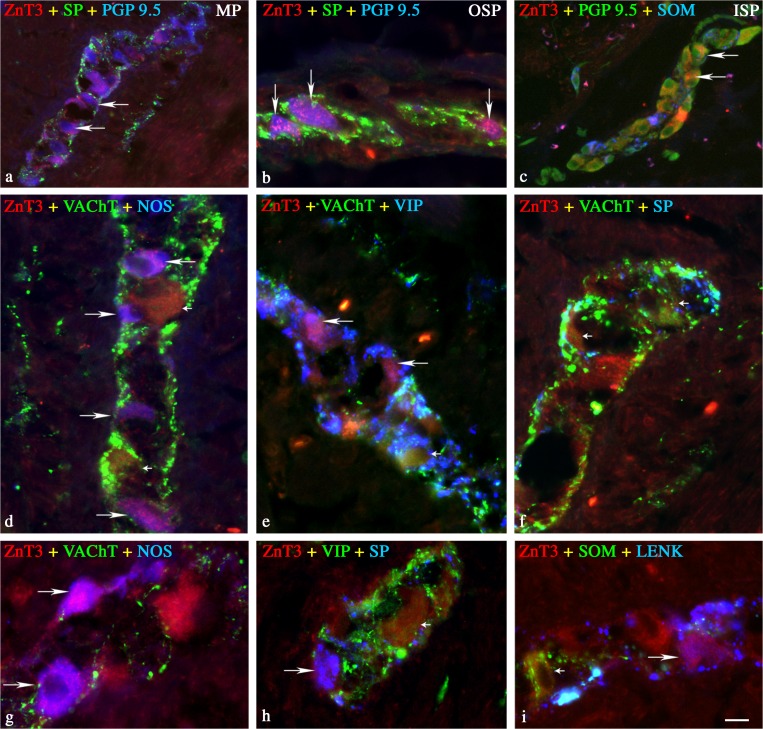
Representative images of ZnT3^+^ neurons located in porcine jejunum. All images are composites of merged images taken separately from blue, red, and green fluorescent channels. **a** Myenteric plexus (*MP*); ZnT3 (*red*) neurons labeled for PGP9.5 (*blue*) but SP (*green*)-immunonegative (*two single arrows*). **b** Outer submucosal plexus (*OSP*); ZnT3 (*red*) neurons labeled for PGP9.5 (*blue*) but SP (*green*)-immunonegative (*three single arrows*). **c** Inner submucosal plexus (*ISP*); ZnT3 (*red*) neurons labeled for PGP9.5 (*green*) but SOM (*blue*)-immunonegative (*two single arrows*). **d–i** ZnT3^+^ neurons located in MP of porcine jejunum. **d** ZnT3 (*red*) neuron labeled for NOS (*blue*) and VAChT (*green*)-immunonegative (*four single arrows*), and ZnT3 (*red*) neurons labeled for VAChT (*green*) but NOS (*blue*)-immunonegative (*two single small arrows*). **e** ZnT3 (*red*) neurons labeled for VIP (*blue*) but VAChT (*green*)-immunonegative (*two single arrows*), and ZnT3 (*red*) neurons labeled for VAChT (*green*) but NOS (*blue*)-immunonegative (*single small arrow*). **f** ZnT3 (*red*) neuron labeled for VAChT (*green*) but SP (*blue*)-immunonegative (*two single small arrows*). **g** ZnT3 (*red*) neuron labeled for NOS (*blue*) but VAChT (*green*)-immunonegative (*two single arrows*). **h** ZnT3 (*red*) neurons labeled for SP (*blue*) but VIP (*green*)-immunonegative (*single arrow*), and ZnT3 (*red*) neurons labeled for VIP (*green*) but SP (*blue*)-immunonegative (*single small arrow*). **i** ZnT3 (*red*) neurons labeled for LENK (*blue*) but SOM (*green*)-immunonegative (*single arrow*), and ZnT3 (*red*) neurons labeled for SOM (*green*) but LENK (*blue*)-immunonegative (*single small arrow*). *Bar* 25 μm

ZnT3^+^ cell bodies were observed in all enteric plexuses within porcine jejunum, i.e., in the MP (located between the longitudinal and circular muscle layers), the OSP (found in the *submucosa*), and the ISP (located on the abluminal side of the *muscularis mucosae*). The proportions of ZnT3^+^ neurons varied depending on the plexus studied, being the largest within the OSP and the smallest in the MP; however, these differences between percentages were not statistically significant (Table 2, Fig. 1a–c). No ZnT3^+^ nerve fibers were observed in the porcine jejunum.

### Co-localization pattern of ZnT3^+^ neurons in porcine jejunum

In all plexuses, the population of ZnT3^+^ neurons could be subdivided into cholinergic (Znt3^+^/VAChT^+^) and non-cholinergic (Znt3^+^/VAChT^−^) neurons. Both cholinergic and non-cholinergic Znt3^+^ cells co-expressed a broad-spectrum of the other substances tested in the present study, but the co-localization patterns were different in each of the plexus studied (Table 2). The only substance that was never co-expressed in ZnT3^+^ neurons was CGRP (Table 2).

#### Myenteric plexus

The MP was the only plexus in which the percentages of cholinergic and non-cholinergic ZnT3^+^ neurons were almost similar; however, even here the cholinergic ZnT3^+^ neurons outnumbered the non-cholinergic ZnT3^+^ cells (Table 2). The great majority of the cholinergic ZnT3^+^ neurons did not co-express any of the tested peptides (Table 2). Among the cholinergic ZnT3^+^ cells that co-expressed additional peptides, the largest population were those containing NOS (Table 2, Fig. 1d, g). In addition, small percentages of the cholinergic ZnT3^+^ neurons co-expressed SOM, VIP, and/or SP (Table 2, Fig. 1e, f). None of the cholinergic ZnT3^+^ neurons was ever immunoreactive for GAL (Table 2). Among the non-cholinergic ZnT3^+^ neurons, almost 50% were devoid of any of the tested peptides (Table 2). However, a huge subpopulation of the non-cholinergic ZnT3^+^ neurons were simultaneously immunoreactive for NOS (Table 2). In addition, small percentages of the non-cholinergic ZnT3^+^ neurons co-expressed SOM, VIP, SP, and/or LENK, and single ZnT3^+^ cells co-expressed NPY (Table 2, Fig. 1h, i). None of the non-cholinergic ZnT3^+^ neurons was ever found to be immunoreactive for GAL or CGRP (Table 2).

#### Outer submucosal plexus

**Fig. 2:**
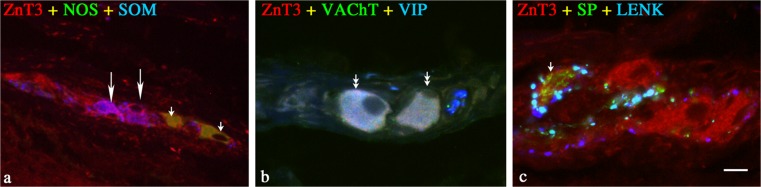
Representative images of ZnT3^+^ neurons located in OSP of porcine jejunum. All images are composites of merged images taken separately from the blue, red, and green fluorescent channels. **a** ZnT3 (*red*) neuron labeled for NOS (*green*) but SOM (*blue*)-immunonegative (*two small single arrows*), and ZnT3 (*red*) neuron labeled for SOM (*blue*) but NOS (*green*)-immunonegative (*two single arrows*). **b** ZnT3 (*red*) neuron labeled for VAChT (*green*) and VIP (*blue*)-immunopositive *(two small double arrows*). **c** ZnT3 (*red*) neuron labeled for SP (*green*) and VIP (*blue*)-immunonegative (*small arrow*). *Bar* 25 μm

In the OSP, the cholinergic ZnT3^+^ cells constituted almost 95% of the total population of ZnT3^+^ neurons (Table 2). Triple-labeling immunofluorescence revealed that a large percentage of the cholinergic ZnT3^+^ neurons in the OSP was also immunoreactive for SOM, and many cells co-expressed VIP and/or SP (Table 2, Fig. 2b). In addition, a small percentage of the cholinergic ZnT3^+^ neurons co-expressed NOS (Table 2), but no immunoreactivity for GAL was observed (Table 2). The non-cholinergic ZnT3^+^ neurons in the OSP formed a small subpopulation of ZnT3^+^ cells, and most of them were devoid of any of the tested peptides (Table 2). Only a small percentage of these neurons co-expressed NOS, SOM, VIP, and/or SP, and single ZnT3^+^ cells co-expressed GAL (Table 2, Fig. 2a). No immunoreactivity for LENK, NPY, or CGRP was observed in these neurons (Table 2, Fig. 2c).

#### Inner submucosal plexus

**Fig. 3:**
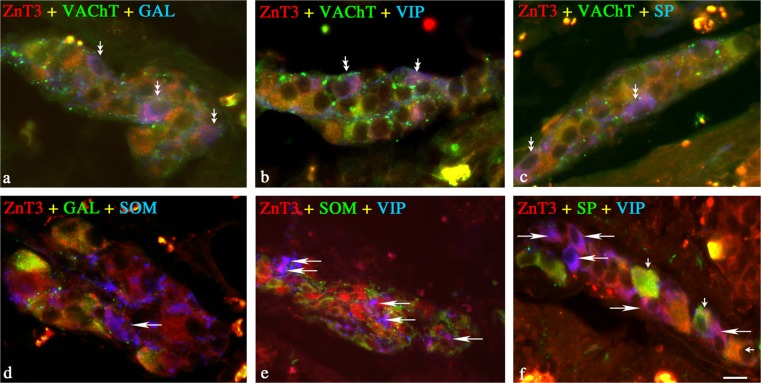
Representative images of ZnT3^+^ neurons located in ISP of porcine jejunum. All images are composites of merged images taken separately from the blue, red, and green fluorescent channels. **a** ZnT3 (*red*) neurons labeled for GAL (*blue*) and VAChT (*green*)-immunopositive (*three double small arrows*). **b** ZnT3 (*red*) neuron labeled for VIP (*blue*) and VAChT (*green*)-immunopositive (*two double small arrows*). **c** ZnT3 (*red*) neurons labeled for VAChT (*green*) and SP (*blue*)-immunopositive (*two double small arrows*). **d** ZnT3 (*red*) neuron labeled for SOM (*blue*) but GAL (*green*)-immunonegative (*single arrow*). **e** ZnT3 (*red*) neurons labeled for VIP (*blue*) but SOM (*green*)-immunonegative (*five single arrows*). **f** ZnT3 (*red*) neuron labeled for VIP (*blue*) but VAChT (*green*)-immunonegative (*five single arrows*), and ZnT3 (*red*) neurons labeled for VAChT (green) but VIP (*blue*)-immunonegative (*three single small arrows*). *Bar* 25 μm (**a–d**, **f**), 100 μm (**e**)

In the ISP, almost all ZnT3^+^ neurons were simultaneously cholinergic (Table 2). Moreover, triple-labeling immunofluorescence revealed that almost half of these cholinergic ZnT3^+^ neurons were also GAL-positive, and that many other cells co-expressed SOM, VIP, and SP (Table 2, Fig. 3a–c). On the other hand, none of the cholinergic ZnT3^+^ neurons was ever immunoreactive for NOS (Table 2). The subpopulation of non-cholinergic ZnT3^+^ neurons in the ISP was even smaller than that in the OSP, and as in the MP and OSP, most of these neurons were devoid of any of the tested peptides (Table 2). Small percentages of the non-cholinergic ZnT3^+^ neurons co-expressed GAL, SOM, VIP, and/or SP (Table 2, Fig. 3d–f). None of the non-cholinergic ZnT3^+^ neurons co-expressed NOS, LENK, NPY, or CGRP (Table 2).

## Discussion

This is the first report providing a detailed description of the distribution, number, and neurochemical characteristics of the ZnT3^+^ neurons in the ENS of porcine jejunum. Numerous cells immunoreactive for Znt3 have been found within all types of jejunal ganglia, a finding generally consistent with previous investigations on human and porcine large intestine (Gonkowski et al. 2009; Gonkowski 2011). The considerable quantity of ZnT3^+^ cells, namely more than 35% of all enteric neurons in each plexus, and the broad-spectrum of active substances that co-localize with this peptide in each plexus suggest that ZnT3 and Zn are important factors within the digestive tract, and that both might be involved in the regulation of various processes in the gut. Moreover, a previous study of porcine large intestine (Gonkowski 2011) has indicated the participation of ZnT3 and Zn in mechanisms of pathological states within the digestive tract. Although the precise function(s) of ZnT3 neurons and Zn are currently unknown, their presence in the enteric ganglia (this study; Gonkowski et al. 2009; Gonkowski 2011) and in the adrenergic and cholinergic sympathetic neurons of the murine peripheral nervous system (Wang et al. 2001a, 2003; Wang and Dahlstrom 2008) suggest that they play an integral role in the function of the gut. The majority of work concerning the role of ZnT3 and of Zn targets has been conducted in the CNS. Previous studies of Zn-containing neurons in the CNS imply that ZnT3 facilitates neuromodulation and/or protects neurons from the cytotoxic action of Zn during pathological processes (Cousins et al. 2006; Danscher et al. 2003; Kim et al. 2000; Palmiter and Huang 2004; Wang et al. 2003; Wenzel et al. 1997). ZnT3 in particular plays an important part in the regulation of Zn levels and is the key protein involved with Zn transport in synaptic vesicles (Danscher et al. 2003; Wenzel et al. 1997). Both ZnT3 and Zn are primarily present in glutamatergic terminals within the CNS (Danscher et al. 2001). However, Zn is also present in GABA- and glycine-containing neurons (Danscher et al. 2001; Birinyi et al. 2001; Wang et al. 2001b). In the hippocampus, Zn is co-released with glutamate from synapses, where it exerts a strong modulatory effect on N-methyl-D-aspartate (NMDA) receptors (Vogt et al. 2000). Moreover, evidence has been presented that Zn can modulate both excitatory and inhibitory neurotransmission (Smart et al. 2004). Excitatory NMDA receptors are directly inhibited by Zn, whereas non-NMDA receptors appear relatively unaffected (Paoletti and Neyton 2007; Smart et al. 2004). Because it is released at many glutamatergic synapses, Zn is likely to be an endogenous allosteric modulator of NMDA receptors (Paoletti and Neyton 2007). Glycinergic (inhibitory) transmission in the CNS might also be affected by Zn, thereby causing potentiation (Betz and Laube 2006; Smart et al. 2004). Low concentrations of Zn potentiate submaximal glycine-induced currents, whereas higher concentrations cause competitive inhibition (Betz and Laube 2006). A point mutation at the murine Glra1 locus, which selectively suppresses Zn potentiation, generates a phenotype that mimics that of patients with hereditary startle disease and thus is indicative of decreased glycinergic inhibition (Betz and Laube 2006). All these data taken together indicate that Zn is a potent modulator of both excitatory and inhibitory neurotransmission within CNS, and so it might play a similar role in ENS. Such a mechanism might in part explain the co-expression of ZnT3 by so many excitatory cholinergic and inhibitory nitrergic intestinal neurons (present study). In particular, cholinergic receptors in the brain might be directly up- or down-regulated by Zn, whereas other cells types might be modulated via various other types of receptors, such as opioid or catecholamine receptors (Frederickson et al. 2005). Since each of the enteric plexuses has its own unique set of functions (Brown and Timmermans 2004; Furness 2006; Shimizu et al. 2008; Timmermans et al. 2001), the possible roles of ZnT3^+^ neurons in each of these plexuses will be discussed separately below.

The MP, which is located between the longitudinal and circular muscle layers, is one of a number of elements that are responsible for the control of digestive motility (Huizinga et al. 2011), and a subpopulation from this plexus also innervates the submucosal plexuses and/or regulates the secretory functions of the gut (Brehmer et al. 1994). Moreover, the long myenteric pathway that activates the submucosal secretomotor neurons projects in parallel with motor and vasodilator reflexes, suggesting that this common pathway coordinates intestinal secretion, blood flow, and motility (Reed and Vanner 2003, 2007). Gut motility is controlled by a subpopulation of cholinergic and nitrergic neurons that mediate the respective contraction and relaxation of the circular and longitudinal muscles (Boeckxstaens et al. 1993; Furness 2006; Lincoln et al. 1997; Porter et al. 1996, 1997; Wood et al. 1999). The majority of ZnT3^+^ myenteric neurons are also immunoreactive for VAChT and/or NOS. Thus, ZnT3 might be present in both neuron populations that act together in co-ordinated reflexes to facilitate smooth muscle contraction (VAChT neurons) and muscle relaxation (NOS neurons), respectively, in the gut. Although we have also found, among the myenteric ZnT3^+^ neurons, cells immunoreactive for various other well-known markers, such as VIP, SOM, and SP, these subpopulations are extremely small and so will not be discussed in the case of MP.

The neurons of the OSP (located near the internal part of circular muscle layer) and ISP (located on the abluminal side of the *muscularis mucosae*) innervate the submucosal blood vessels and regulate the secretion and intrinsic sensory pathways of the gut in response to the contents of the lumen (Brehmer et al. 2010). A subpopulation of the OSP neurons might also supply the circular muscle layer of the gut (Scheuermann and Timmermans 1993). The results of the present study suggest that almost half of all neurons located in the OSP and ISP is ZnT3-positive, and almost all of the ZnT3^+^ neurons in both these plexuses are simultaneously immunoreactive for VAChT. Such a large population of ZnT3^+^/VAChT^+^ neurons indicates that ZnT3 is engaged in various excitatory functions in the OSP and ISP, including muscle contraction in the *muscularis mucosa* and the increased activity of the mucosal glands (Cooke 2000). Many of the ZnT3^+^ neurons in the OSP and ISP have been found to be immunoreactive for SOM, VIP, and/or SP; this is consistent with previous studies concerning ZnT3^+^ neurons in the porcine large intestine (Gonkowski 2011). SOM exerts many effects in the ENS; these include the inhibition of peristalsis (Grider et al. 1987), the regulation of blood flow in the intestines (Li et al. 1996), the inhibition of gastrin, cholecystokinin, and VIP (Foong et al. 2010; Low 2004), and the inhibition of extrinsic afferent sensory neuron activity (Furness 2012; Hasler et al. 1993; Plourde et al. 1993). However, the role of ZnT3 in these processes is obscure at present. VIP is strongly co-expressed in ZnT3^+^ neurons of both submucosal plexuses, but especially in the ISP. This is congruent with the finding that submucosal VIP neurons are responsible for increasing secretory activity at mucosal glands in the small intestine (Olsson and Holmgren 2001). SP is also an important co-transmitter of cholinergic excitatory motor neurons, and so unsurprisingly, many of the ZnT3^+^ neurons immunoreactive for VAChT also co-express SP (Furness 2006; Shimizu et al. 2008). In the OSP and ISP, neurons using SP as their neurotransmitter are thought to be involved mostly in the regulation of the transport and/or secretion of H_2_O and/or electrolytes (Shimizu et al. 2008; Keast et al. 1985), and so ZnT3 might also be engaged in all these processes. We should add that SP, in both these plexuses, might also be a neurotransmitter and/or neuromodulator of interneurons or cells supplying intestinal blood vessels (Shimizu et al. 2008). The last two substances which are worth mentioning here, are GAL and CGRP. GAL is strongly co-expressed by ZnT3^+^ neurons in the ISP, whereas such cells are extremely rare in the OSP (almost half of the ZnT3^+^ neurons in the ISP co-express GAL). A similar pattern of GAL co-expression in ZnT3^+^ neurons has also been observed in the intramural ganglia of the porcine large intestine (Gonkowski 2011). GAL is often expressed in the ISP by neurons that are engaged in the regulation of the intestinal secretion (Furness 2006) and/or neurotransmitter secretion from other intestinal neurons (Piqueras et al. 2004; Sarnelli et al. 2004). The extensive co-localization of ZnT3and GAL in the ISP neurons suggests that ZnT3 and Zn are involved in some of these processes. CGRP is a marker peptide for Dogiel type II neurons, which are putative intrinsic primary afferent neurons (Timmermans et al. 1997; Wolf et al. 2007). However, some recent studies have shown CGRP-positive neurons displaying a distinctly different morphology (Wolf et al. 2007). Although CGRP-containing neurons have been observed in the present study, and although most of them have the features of Dogiel type II neurons, these cells are ZnT3-negative. Thus, detectable ZnT3 immunoreactivity might not occur in intestinal intrinsic sensory neurons.

In conclusion, ZnT3^+^ neurons have been identified in enteric ganglia of the porcine jejunum. A broad-spectrum of neuroactive substances has been found to co-localize with ZnT3, suggesting that ZnT3 neurons are involved in many of the functional processes throughout the length of the jejunum. Further studies are required to elucidate the role of these neurons during pathological states of the jejunum.
